# Immunostaining of Lgr5, an Intestinal Stem Cell Marker, in Normal and Premalignant Human Gastrointestinal Tissue

**DOI:** 10.1100/tsw.2008.148

**Published:** 2008-11-23

**Authors:** Laren Becker, Qin Huang, Hiroshi Mashimo

**Affiliations:** ^1^ Beth Israel Deaconess Medical Center, Boston, London, UK; ^2^ VA Boston Healthcare System, Boston, London, UK; ^3^ Harvard Medical School, Boston, London, UK

**Keywords:** Lgr5, stem cell, cancer, colon, small intestine, adenoma, Barrett's esophagus, CD133, tumorigenesis, stem cell niche

## Abstract

Lgr5 has recently been identified as a murine marker of intestinal stem cells. Its expression has not been well characterized in human gastrointestinal tissues, but has been reported in certain cancers. With the increasing appreciation for the role of cancer stem cells or tumor-initiating cells in certain tumors, we sought to explore the expression of Lgr5 in normal and premalignant human gastrointestinal tissues. Using standard immunostaining, we compared expression of Lgr5 in normal colon and small intestine vs. small intestinal and colonic adenomas and Barrett's esophagus. In the normal tissue, Lgr5 was expressed in the expected stem cell niche, at the base of crypts, as seen in mice. However, in premalignant lesions, Lgr5^+^ cells were not restricted to the crypt base. Additionally, their overall numbers were increased. In colonic adenomas, Lgr5^+^ cells were commonly found clustered at the luminal surface and rarely at the crypt base. Finally, we compared immunostaining of Lgr5 with that of CD133, a previously characterized marker for tumor-initiating cells in colon cancer, and found that they identified distinct subpopulations of cells that were in close proximity, but did not costain. Our findings suggest that (1) Lgr5 is a potential marker of intestinal stem cells in humans and (2) loss of restriction to the stem cell niche is an early event in the premalignant transformation of stem cells and may play a role in carcinogenesis.

## INTRODUCTION

Mounting evidence suggests that cancer is a disease of stem cells[1,2,3]. As first recognized in hematologic malignancies, and more recently in solid tumors, cancers contain a subpopulation of cells termed "tumor-initiating cells" or "cancer stem cells" (CSCs) that have the potential to generate the entire tumor hierarchy[4,5,6,7,8]. The origin of these CSCs is unclear; whether they arise from mutant stem cells and, if so, whether there are early signs that a population of stem cells is "unstable" and prone to transformation. One of the difficulties in addressing these questions has been the paucity of markers for native stem cells.

Barker et al. recently demonstrated that Lgr5 (also called GPR49), an orphan G-protein coupled receptor and Wnt target gene, is a putative marker of murine intestinal stem cells[9]. They identified a subpopulation of $Lgr5^+$ stem cells at the base of crypts in mouse small intestine and colon. Using mice derived from crossing a knock-in Lgr5-GFP fusion allele with a tamoxifen-induced lacZ reporter (mediated by Cre-recombinase), they performed clonal replacement studies and demonstrated that these $Lgr5^+$ cells yielded "blue ribbons" of progeny cells that extended up the crypt-villus axis. These "ribbons" were maintained even at 60 days postinduction and contained the entire hierarchy of crypt-villus cells, including enterocytes, goblet cells, paneth cells, and enteroendocrine cells, thereby fulfilling the criteria of "stemness" with long-term maintenance and multipotency.

Using an antibody to human Lgr5, we sought to investigate the presence of Lgr5-immunoreactive stem cells in the crypts of human colon and small intestine. We next examined the pattern of immunostaining of Lgr5 in premalignant lesions, including colonic adenomas, small intestinal adenomas, and Barrett's esophagus (BE), a specialized intestinal metaplasia of the esophagus. Finally, we compared expression of Lgr5 with CD133, a known tumor-initiating cell marker[6,7], to assess whether they identify the same population of cells in colonic adenomas.

## MATERIALS AND METHODS

### Tissue Samples

The study was carried out on paraffin-embedded, formalin-fixed human tissue samples, including surgical resections and endoscopic biopsy specimens. Patient medical records were reviewed and gastrointestinal histopathology was correlated with each specimen. Normal colon and small intestine tissue, five colonic adenomas, two duodenal adenomas, and 10 BE specimens were randomly selected and used in the study. The colonic adenomas were low-grade adenomas. One adenoma was greater than 2 cm diameter and the other four were approximately 1 cm in diameter. The duodenal adenomas were removed from patients without familial adenomatous polyposis. The study protocol was approved and in compliance with the Research and Development Committee of the Boston VA Healthcare System.

### Immunohistochemistry

Each paraffin block was cut at 4-µm in thickness. Sections were mounted, deparaffinized in xylene, and rehydrated through descending concentrations of ethanol. Antigen retrieval was performed using citrate buffer (Vector Lab, Burlingame, CA) heated in a pressure cooker for 5 min and then cooled to room temperature. Blocking of endogenous peroxidases was accomplished by incubating sections in 3% hydrogen peroxide (Sigma, St Louis, MO) for 5 min. Lgr5 polyclonal antibodies (MBL International Co., #LS-A1235 at 1:50 dilution and #LS-A1232 at 1:200 dilution), monoclonal Ki67 antibody (Dako, MIB-1, #M7240 at 1:100 dilution), and monoclonal CD133 antibody (Abcam, #ab27699 at 1:250 dilution) were incubated with sections overnight at $4^o$. Immunostaining was performed by using Envision+ System and diaminobenzidine (DAB) visualization (Dako, Carpinteria, CA) according to the manufacturer's instructions. Sections were counterstained with hematoxylin for 1 min, rinsed in water, dehydrated in ascending concentrations of ethanol followed by clearance with xylene, and cover slipped permanently for light microscopy.

Coimmunofluorescence was performed using TSA Cya-5 Tyramide reagent pack (PerkinElmer, Waltham, MA, #SAT705A) for Lgr5 and Alexa Fluor 568-conjugated antimouse IgG (Invitrogen, Carlsbad, CA, #A-11004) as secondary for Ki67 and CD133. Nuclei were stained using SYBR Green 1 Nucleic Acid Gel Stain (Fisher Scientific, Pittsburgh, PA, #BMA50513) prior to mounting. Negative controls were carried out with the same procedure without primary antibody.

## Imaging

Bright-field micrographs were captured on a Leica DM 2000 microscope using a RT Color Spot camera (Diagnostic Instruments). Confocal images were captured using a Nikon C1 confocal microscope.

## RESULTS

Immunostaining revealed expression of Lgr5 in a subpopulation of epithelial cells at the crypt base of normal human colon and small intestinal mucosa, similar to the pattern of expression in mouse colon shown by Barker et al.[9] (Fig. 1). The number of positive cells per crypt varied from a single cell to several, but many crypts showed no immunostaining. The cellular staining was membranous and cytoplasmic in distribution. A similar staining pattern was seen using two different polyclonal Lgr5 antibodies, one of which recognizes epitopes on the second cytoplasmic domain (#LS-A1235) (Fig. 1A) and the other the N-terminal extracellular domain (#LS-A1232) (Fig. 1B). The antibody to the cytoplasmic domain tended to give higher-intensity staining. There were scattered immunoreactive cells noted in the lamina propria that may represent real staining of hematopoietic cells (not seen on negative controls). Lgr5 has previously been found to express in bone marrow based on tissue mRNA expression studies[10].

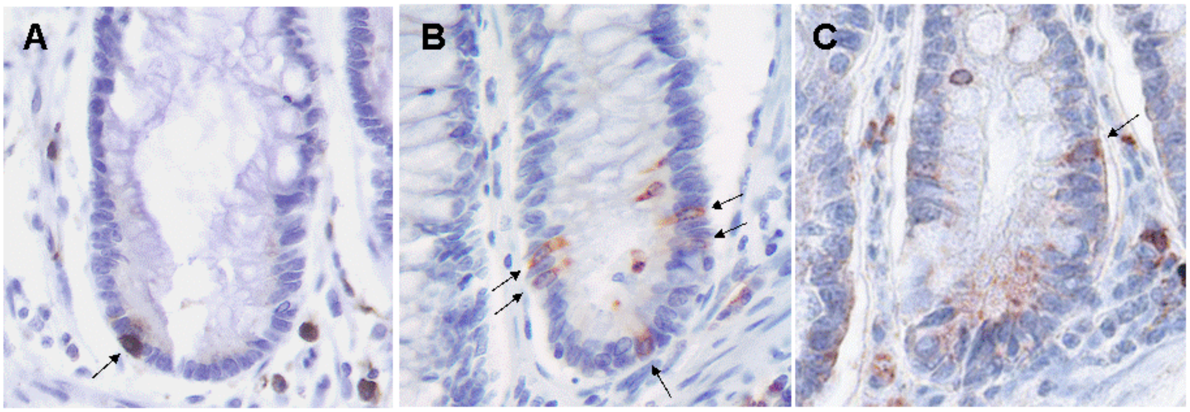

**FIGURE 1.** Lgr5 immunostaining is restricted to cells at the base of crypts (arrows) in normal human colonic and small intestinal mucosa. Lgr5 antibody to the second cytoplasmic domain in (A) colon (40×) and (C) small intestine (40×), and Lgr5 antibody to the N-terminal extracellular domain in (B) colon (40×).

Immunostaining of colonic adenomas was also similar to the pattern seen in the adenomas from $APC^{min}$ mice in the Barker paper[9] (Fig. 2). In four of five adenomas, we found clusters of $Lgr5^{+}$ cells in a background of negative cells. In striking contrast to normal crypts, Lgr5 staining was not restricted to crypt base in adenomas (Fig. 2A). In fact, the most common staining pattern was at the luminal surface with sharp transitions between the immunoreactive and nonimmunoreactive cell borders along the crypt axis (Fig. 2B). Isolated $Lgr5^{+}$ cells at the crypt base as seen in normal colon were rare. Instead, Lgr5-immunoreactive epithelial cells were clustered, forming patches. Their relative numbers were increased per crypt compared with normal colon. When coimmunofluorescence was performed with Lgr5 (blue) and the proliferation marker Ki67 (red), $Lgr5^{+}$ cells were less likely to stain for Ki67 than neighboring cells (Fig. 2C). This was confirmed on adjacent sections with immunohistochemistry (Fig. 2D and E). Overall, there appeared to be greater Ki67 staining at the adenoma surface than at the crypt base.

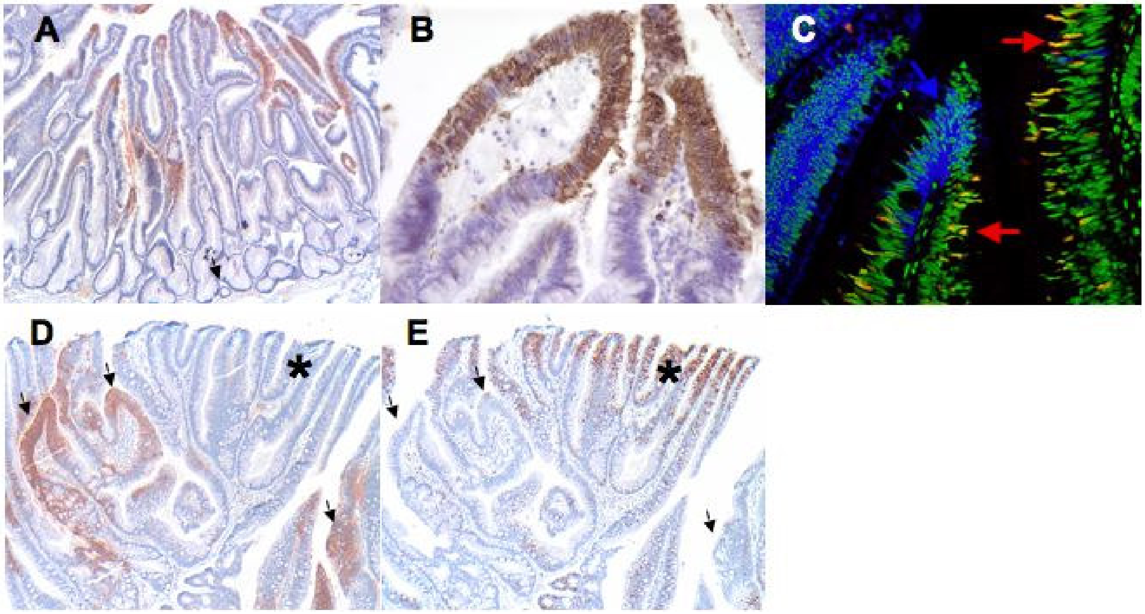

**FIGURE 2**. Aberrant staining pattern of Lgr5 in colonic adenoma. (A) Patchy staining of Lgr5 (10×). Minimal staining was observed at the crypt base (arrow). (B) Staining of Lgr5 at surface with a sharp transition relative to nonstaining cells (20×). (C) Coimmunofluorescence of Lgr5 with Cy5 (blue) and Ki67 with Alexa Fluor 568 (red) (20×). Nuclei (green) are stained with SYBR Green Nucleic Acid Stain. There is minimal Ki67 immunostaining in the $Lgr5^+$ cells (blue arrow) compared with neighboring $Lgr5^-$ cells (red arrows). (D) Adjacent section stained for Lgr5 (10×). (E) Adjacent section stained for Ki67 (10×). Notice the relatively decreased staining of Ki67 in $Lgr5^+$ cells (arrows) and relatively increased staining of Ki67 at the surface where Lgr5 staining is minimal (asterisks).

Immunohistochemistry was performed on other premalignant lesions including 10 BE samples and two duodenal adenomas. Lgr5 staining was observed in seven of 10 BE specimens and in both intestinal adenomas. Staining patterns similar to those of colonic adenomas were observed (Fig. 3A–D). The $Lgr5^+$ cells were seen in clusters, with sharp transitions relative to the adjacent negative epithelial cells (Fig. 3B and D). They could be found at the crypt base, but were not restricted there, and were often present at the surface (Fig. 3A and D).

Lgr5 has previously been shown to be expressed in colon, ovarian, and hepatocellular carcinomas[11,12]. We were able to extend this finding to colon cancer. Additionally, we have found intense Lgr5 immunostaining in BE-associated dysplastic lesions, esophageal adenocarcinoma (EAC), and gastric carcinoma (data not shown). Given its role as an intestinal stem cell marker, Lgr5 gene expression in cancer has led to speculation regarding its potential as a CSC marker[13,14,15]. To explore this possibility further, we evaluated whether Lgr5 costains the same population of cells in colonic adenomas as CD133, a tumor-initiating cell marker in colon cancer[6,7]. Using coimmunofluorescence and immunohistochemistry of adjacent sections, we found that Lgr5 and CD133 identified two distinct, mutually exclusive populations of neoplastic cells within the same adenoma (Fig. 4A–C).

## DISCUSSION

The existence of native stem cells in gut epithelium has been postulated for decades[16,17,18]. However, proof of their existence has been hampered by lack of a suitable marker(s). This has changed following the discovery by Barker et al. that Lgr5 labels a subpopulation of epithelial cells at the base of crypts in mouse small intestine and colon that fulfills the criteria of "stemness": multipotency and prolonged self-renewal. Using standard immunostaining, we showed that $Lgr5^+$ cells are present at the base of normal

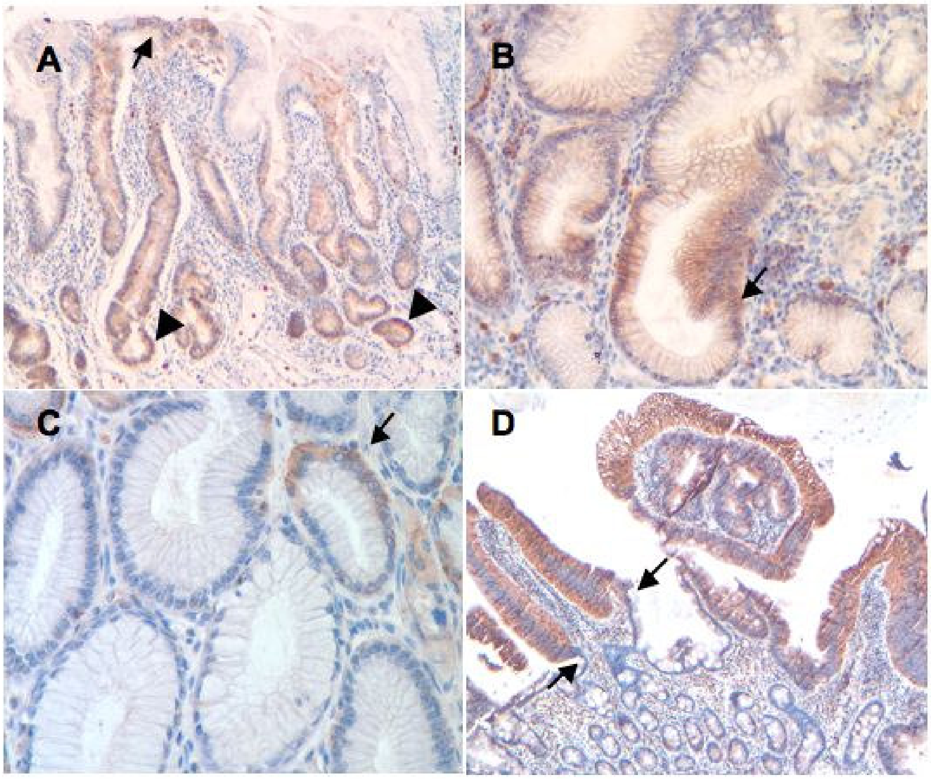

**FIGURE 3**. Aberrant staining pattern of Lgr5 in other premalignant lesions. (A) Staining of Lgr5 in BE (10×). There is a patchy distribution of expression, with staining at the crypt base (arrowheads) and surface (arrow). (B) Staining of Lgr5 in BE (20×). There is a sharp transition between Lgr5$^+$ cells and the negative cells at the crypt base (arrow). (C) Staining of Lgr5 in columnar epithelium in distal esophagus (20×). On cross-sections of crypts, Lgr5 stained half of the crypt (arrow). (D) Staining of Lgr5 in duodenal adenoma (10×). Lgr5 staining is seen preferentially at the surface with sharp transitions (arrows).

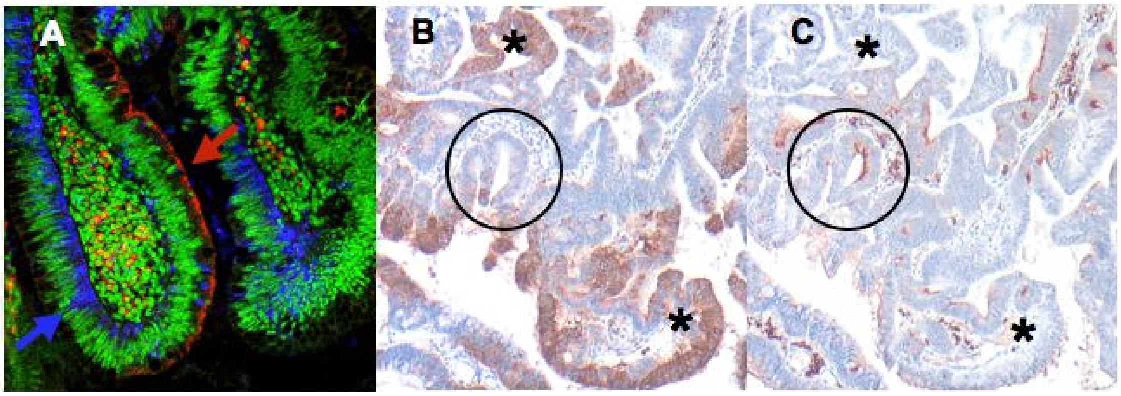

**FIGURE 4.** Coimmunostaining of Lgr5 and CD133 in colonic adenoma identifies distinct populations of cells. (A) Coimmunofluorescence of Lgr5 with Cy5 (blue) and CD133 with Alexa Fluor 568 (red) (40×). Nuclei (green) are stained with SYBR Green. Immunostaining of Lgr5 (blue arrow) and CD133 (red arrow) reveals distinct subpopulations of cells. (B) Adjacent section stained for Lgr5 (10×). (C) Adjacent section stained for CD133 (10×). The two subpopulations of cells were often in close proximity, but were distinct and mutually exclusive (circles and asterisks).

human colonic and small intestinal crypts. This is in a distribution similar to that reported in mice and consistent with the known location of the stem cell niche[9,19,20]. We found similar Lgr5 immunostaining patterns using two antibodies that recognize different epitopes on the Lgr5 protein molecule, suggesting that the staining is not artifact. Lgr5 is an important marker to examine the role of intestinal stem cells in human diseases, and particularly in cancer.

The stem cell niche has been known to control the balance of proliferation and differentiation along the crypt-villus axis tightly through a variety of regulatory factors, including Wnt, hedgehog, bone morphogenetic protein, Eph, and Notch[21,22,23,24,25,26]. It has been speculated that changes in the interaction between a stem cell and its niche may play a role in tumorigenesis[14,27,28]. When we compared the staining patterns of Lgr5 in normal colon with those in premalignant conditions, we found that the most striking feature was the loss of restriction of $Lgr5^+$ cells to the stem cell niche at the crypt base. Additionally, “outside” of the stem cell niche, the pool of $Lgr5^+$ cells appears to have expanded. Given that Lgr5 is a known target gene of Wnt signaling, it is intriguing to speculate that in moving out of the stem cell niche, the regulatory milieu has shifted in favor of Wnt signaling, leading to increased symmetric divisions and expansion of the stem cell population. An interesting observation in our study is that even outside the stem cell niche microenvironment, $Lgr5^+$ cells did not demonstrate significant staining for Ki67, compared with neighboring (possibly daughter) neoplastic cells. This suggests that the $Lgr5^+$ cells retain some intrinsic regulation that maintains them in a relatively quiescent state irrespective of the niche microenvironment. Our findings seem to support a model of tumorigenesis, whereby movement of stem cells out of the stem cell niche is an early event in the process. It will be interesting to examine whether changes in the balance of cell proliferation regulatory factors in premalignant lesions might account for the stem cell patterns we observe.

Our findings seem to favor a “top-down” vs. “bottom-up” model of adenomatous formation[29,30]. This ongoing debate revolves around location of the mutant stem cells that are driving adenomatous growth. In the “bottom-up” theory, the mutant stem cells expand from the crypt base, leading to clonal conversion and crypt fission[14,29]. In the “top-down” theory, a population of stem cells or progenitor cells in the intercryptal zones at the surface is driving tumor growth downward[30,31]. While we occasionally observed patches of $Lgr5^+$ cells at the crypt base in colonic adenomas, it was more common to find them at the surface than at the base. These Lgr5 immunoreactive clusters appeared isolated, with sharp transitions to the adjacent $Lgr5^-$ neoplastic cells and no extension to the crypt base. This pattern would favor the “top-down” model for tumor expansion. There are, however, several limitations regarding interpretation and generalization of our findings. For example, our adenomas tend to be large (approximately 1 cm in size) and it has been observed that larger adenomas may demonstrate “top-down” expansion, while early adenomas or microadenomas grow by crypt fission in a “bottom-up” fashion[29]. Second, we have a small sample size and, therefore, we may miss the full disease spectrum for adenoma tumorigenesis. Future studies should also evaluate whether the Lgr5 staining pattern varies between different types of adenomas, such as sporadic vs. hereditary (familial adenomatous polyposis and serrated adenomas associated with Lynch Syndrome), microsatellite instable vs. microsatellite stable, serrated adenoma vs. tubular adenoma, etc.

BE is believed to be a condition driven by stem cells. However, the origin of these stem cells has yet to be elucidated[32]. Some have speculated that the origin of these stem cells is within the esophagus[32,33]. Others have proposed that the stem cells may be derived from bone marrow progenitor cells[34]. Here we have demonstrated Lgr5 immunoreactive epithelial cells as a subpopulation of epithelial cells in BE. We found the Lgr5 staining pattern to be similar to that in colonic and duodenal adenomas, with loss of restriction to the crypt base. Lgr5 may be a marker for the elusive stem cells of BE. It will be interesting to seeif Lgr5 immunoreactivity identifies cells predisposed to progression towards advanced dysplastic lesions and EAC.

In addition to premalignant lesions, Lgr5 has been shown to be expressed in cancers, including colon, ovarian, and hepatocellular cancers[11,12]. We confirmed this finding in colon cancer and have also found Lgr5 expression in BE-associated dysplastic lesions, EAC, and gastric cancer (data not shown). With the increasing appreciation of CD133 as a marker for tumor-initiating cells in colon cancer, we sought to explore the relationship of this marker with Lgr5. We found that $Lgr5^+$ cells are distinct from $CD133^+$ cells in the same adenoma. The CD133-expressing cells were often in close proximity to the $Lgr5^+$ cells, suggesting that they may be progeny of the Lgr5 cells. A recent study raises new questions regarding CD133 as a marker for tumor-initiating cells in colon cancer[35]. The study found that the $CD133^-$ subset of cells taken from metastatic colon cancer lesions were not only capable of forming tumors, but did so at a faster rate than the $CD133^+$ subset. Additionally, they found that CD133 was expressed ubiquitously in normal colonic epithelium. This suggests that the subpopulation of $CD133^+$ cells in cancer is derived from a differentiated cell population and not from native stem cells. We may speculate that Lgr5 is a marker for CSCs in colon cancer, possibly better than CD133. Given that Lgr5 is a cell surface receptor, $Lgr5^+$ cells should be amenable to sorting, thus allowing orthotopic transplantation tumorigenicity assays[4,5,6,7,8] (injection into SCID mice) to be performed in the future.

Prior studies of cancer stem cells have largely emphasized functional tumorigenicity assays, in which tumor cells are dispersed. Native expression in cancer and premalignant tissue has been poorly characterized. The patchy distribution of Lgr5 expression may be a feature common to other CSCs. It may account for the wide variability in the frequency of CSCs found in different tumors (the fraction of $CD133^+$ tumor cells ranged from 1.8 to 24.5% of total tumor cells in one study[6]). It also likely reflects the heterogenicity of many solid tumors.

Further studies will require larger sample sizes to evaluate the full spectrum of disease. Additionally, assessment of stem cell clonality could be accomplished by microdissections with mutational analysis. Nonetheless, we believe that our observations regarding Lgr5 immunostaining offer important insights into gastrointestinal tumorigenesis mechanisms.

## CONCLUSION

We have identified a subpopulation of $Lgr5^+$ cells at the base of crypts in normal human colonic and small intestinal mucosa in a similar location to that seen in the mouse, which presumably represents intestinal stem cells. We have found the staining pattern of Lgr5 to differ between normal tissue and premalignant lesions of colon, small intestine, and esophagus, most notably with loss of restriction of $Lgr5^+$ cells to the crypt base. This observation suggests that movement of stem cells out of their "stem cell niche" is an early event in tumorigenesis.

## ACKNOWLEDGMENTS

Sources of support include the VA Merit Research Award and T32 DK 07760 "Research Training in Gastroenterology" institutional grant through Beth Israel Deaconess Medical Center (Program Director: J. Thomas Lamont, M.D.).
